# Biclonal plasma cell leukemia associated with a complex karyotype, TP53 deletion, and high‐risk disease

**DOI:** 10.1002/jha2.159

**Published:** 2020-12-21

**Authors:** Wei Xie, Guang Fan

**Affiliations:** ^1^ Department of Pathology Oregon Health & Science University Portland Oregon

**FIGURE 1:**
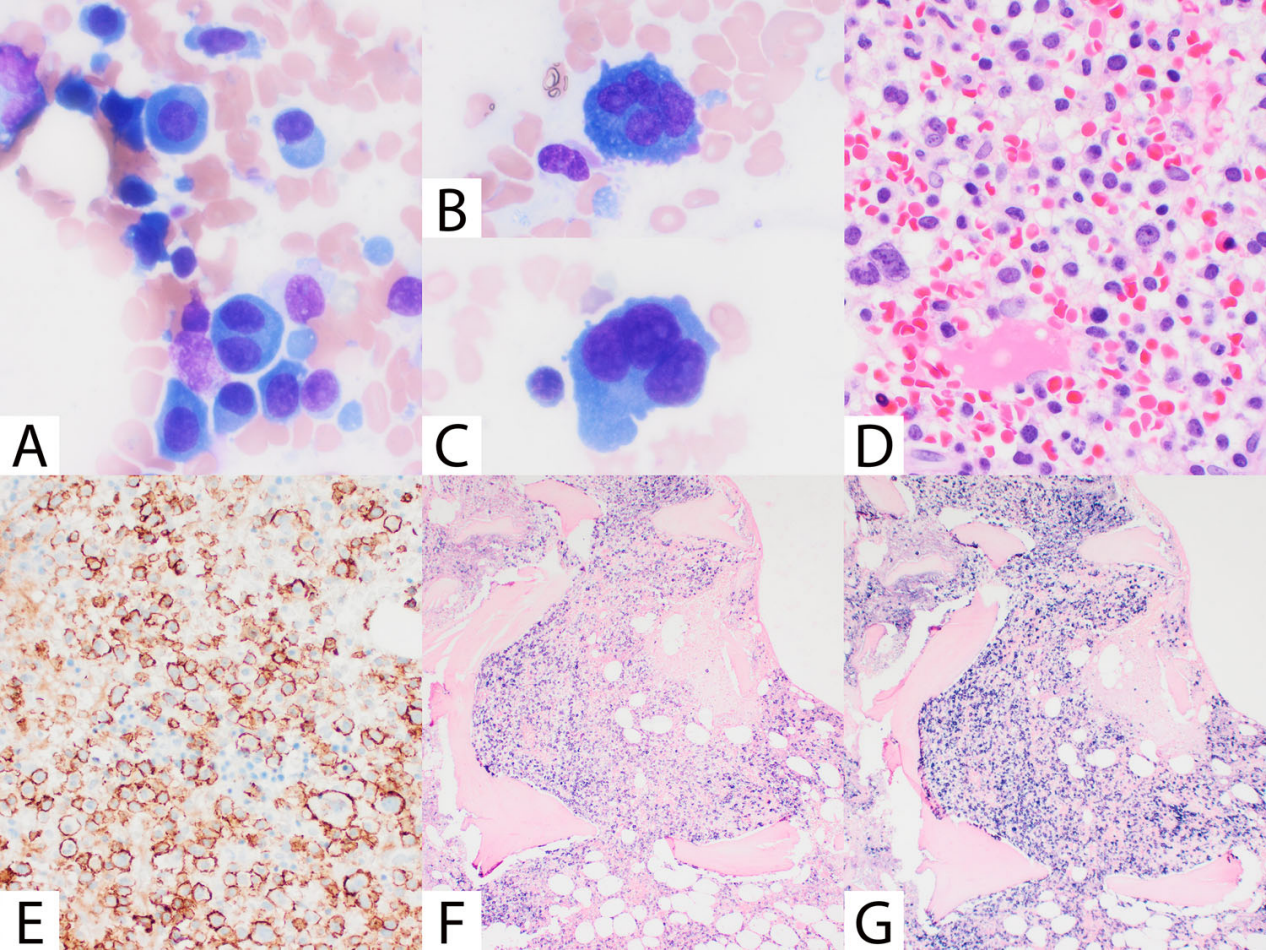
Morphologic and immunophenotypic features of the case with biclonal plasma cell leukemia

A 47‐year‐old man was presented with progressive back pain, rib fracture, anemia, and acute kidney injury. Initially, the peripheral blood showed 40% circulating plasma cells (3.6 × 109/L). Serum immunofixation revealed only lambda light chain monoclonal protein. A diagnosis of plasma cell leukemia was rendered, and the patient was treated with bortezomib, cyclophosphamide, and dexamethasone. The patient was referred to our institute for an autologous stem cell transplant. On the current bone marrow (BM) aspirate, the atypical plasma cells are intermediate to large sized, with occasional binucleation or multinucleation (Figure 1, panels A‐C). The BM biopsy demonstrated hypercellular marrow involved 80% of plasma cells (Figure 1, panel E). The plasma cells are positive for CD138 (Figure 1, panel E), CD56, CD117, p53, cyclinD1, kappa, and lambda light chains (Figure 1, panel F: kappa ISH; Figure 1, panel G: lambda ISH). Fluorescence in situ hybridization (FISH) studies performed on the sorted CD138‐positive cells showed IGH/CCND1 translocation, TP53 deletion, and 1p loss. Conventional cytogenetics showed a complex karyotype. The 220‐gene NGS panel revealed *TP53* p.C124R mutation.

Biclonal plasma cell leukemia/myeloma with only lambda light chain by serum immunofixation is extremely unusual. This phenomenon has been rarely reported in the literature. This case is associated with complex karyotype, *TP53* deletion, and high‐risk disease. The dual expression of kappa and lambda light chain may cause a diagnostic challenge.

